# Neutrophil-to-Lymphocyte Ratio and Prognostic Nutritional Index Are Predictors for Overall Survival after Primary Pancreatic Resection of Pancreatic Ductal Adenocarcinoma: A Single Centre Evaluation

**DOI:** 10.3390/cancers16162911

**Published:** 2024-08-22

**Authors:** Danilo Hackner, Susanne Merkel, Andreas Weiß, Christian Krautz, Georg F. Weber, Robert Grützmann, Maximilian Brunner

**Affiliations:** 1 Department of General and Visceral Surgery, Friedrich-Alexander-University (FAU) Erlangen-Nuremberg, 91054 Erlangen, Germany; susanne.merkel@uk-erlangen.de (S.M.); andreas.weiss@uk-erlangen.de (A.W.); christian.krautz@uk-erlangen.de (C.K.); georg.weber@uk-erlangen.de (G.F.W.); robert.gruetzmann@uk-erlangen.de (R.G.); maximilian.brunner@uk-erlangen.de (M.B.); 2 Department of Anaesthesiology, LMU University Hospital, LMU Munich, 81377 Munich, Germany

**Keywords:** pancreatic ductal adenocarcinoma, pancreatic resection, overall survival, biomarkers, neutrophil-to-lymphocyte ratio, prognostic nutritional index

## Abstract

**Simple Summary:**

Inflammation-based prognostic markers have been recognized as useful in a range of oncologic conditions. Given the high mortality rate associated with pancreatic ductal adenocarcinoma (PDAC), the demand for accurate prognostic tools is significant. Nonetheless, the literature still shows conflicting results regarding the effectiveness of the various available scoring systems. Our data confirm that NLR and PNI are useful biomarkers regarding overall survival in PDAC. Especially in advanced disease stages, they seem to be of considerable benefit as easily accessible tools for prognosis prediction.

**Abstract:**

Purpose: Prognostic inflammation-based parameters have been reported as useful tools in various oncologic diseases. Pancreatic ductal adenocarcinoma (PDAC) is characterized by a high mortality rate, making reliable prognostic markers highly desirable. However, there is still inconsistency in the literature regarding the efficacy of the different available scores. Methods: A total of 207 patients, who underwent primary resection of PDAC from January 2000 to December 2018 at the University Hospital of Erlangen, were included in this retrospective single-center study. Different biomarkers, including the preoperative neutrophil–lymphocyte ratio (NLR), the platelet–lymphocyte ratio (PLR), the c-reactive protein (CRP)–albumin ratio (CAR), the lymphocyte–CRP ratio (LCR), the prognostic nutritional index (PNI) and the modified Glasgow prognostic score (mGPS) were analyzed for their ability to predict overall survival (OS). Results: In our cohort, the median overall survival was 20.7 months. Among the investigated biomarkers, NLR and PNI were identified as independent prognostic markers (Hazard Ratio (HR) 1.6 (1.0–2.5), *p* = 0.048 and HR 0.6 (0.4–0.9), *p* = 0.018), whereas PLR, CAR, LCR and mGPS did not reach significance in the multivariate analysis. Subgroup analysis revealed that the prognostic value of NLR and PNI is particularly evident in locally advanced tumor stages (pT3/4 and pN+). Conclusions: The NLR and PNI could serve as valuable tools for estimating prognosis in patients with PDAC undergoing pancreatic resection in curative intention, especially in locally advanced tumor stages. However, conflicting results in the current literature highlight the need for further prospective studies to validate these findings.

## 1. Introduction

Pancreatic ductal adenocarcinoma (PDAC) is the third most common cause of cancer-related deaths worldwide, accounting for 4.6% of all cancer mortalities [1,2]. The 5-year relative survival is amongst the lowest of all cancers at 42%, even when diagnosed in localized stages [2]. The aggressive nature of pancreatic carcinoma, which often leads to rapid metastasis even in patients who undergo primary resection, contributes to its persistently poor prognosis [3]. Despite improvements in surgical and perioperative treatments over recent decades, patients with PDAC face high mortality and recurrence rates, even after a margin-negative resection without residual tumor (R0-resection) [4]. In the era of precision medicine, where optimized and individualized treatment is the goal, there is a need for prognostic markers to help tailor an appropriate treatment strategy for each individual patient [5,6].

Numerous studies have explored predictive biomarkers for better patient stratification, but there remains widespread disagreement about the optimum score for PDAC. Various laboratory values have been proposed to predict PDAC prognosis, differing from traditional TNM classification of malignant tumors as published by the Union for International Cancer Control (UICC) [7] or molecular biological characteristics, as they can be applied preoperatively. These laboratory markers include the neutrophil–lymphocyte ratio (NLR), the platelet–lymphocyte ratio (PLR), the c-reactive protein (CRP)–albumin ratio (CAR), the lymphocyte–CRP ratio (LCR), the prognostic nutritional index (PNI) and the modified Glasgow prognostic score (mGPS), calculated using albumin and CRP [8]. Among these, NLR is the most widely investigated inflammatory parameter in cancer patients [9]. These scores are based on the increasing understanding of the link between neoplasia and inflammation. Inflammation in the tumor microenvironment supports processes like tumor development, angiogenesis, metastasis as well as an altered response to hormones and chemotherapeutics [10]. Thrombocytosis has often been described as correlating with poor survival, especially due to its role in metastasis [11]. Conversely, lymphocytes have beneficial effects on tumor cells, slowing tumor growth and enhancing responses to cytoreductive treatments [12,13]. Thus, elevated inflammatory parameters and thrombocytosis might correlate with poorer prognosis, while high lymphocytes levels indicate a favorable outcome.

However, current studies on predictive biomarkers in PDAC show inconsistent results [14,15,16,17,18]. The primary aim of this study was to evaluate the usefulness of various scores in patients undergoing curative pancreatic resection in terms of overall survival (OS) to strengthen the clinical applicability in PDAC and facilitate routine clinical practice.

## 2. Patients and Methods

We included 207 adult patients who underwent primary resection of PDAC between 1 January 2000 and 31 December 2018 at the university hospital of Erlangen, Germany. Based on the available diagnostics, all pancreatic malignancies were deemed primarily resectable according to our interdisciplinary tumor board. Patients who had received neoadjuvant chemo- or radiochemotherapy were excluded from the study.

Patients’ clinical data, including laboratory data, were retrieved from the clinical information system. The Erlangen Cancer Registry of the Department of Surgery provided survival and pathological data. Histopathological details were described according to the TNM classification. Morbidity was assessed using the Clavien–Dindo classification, with grades III, IV and V defined as major morbidity [19]. Patients’ mean follow-up time was 33.6 month [range 0–198 months].

The ethic committee of the Friedrich–Alexander University Erlangen–Nuremberg approved this study (22-165-Br).

### 2.1. Study Design

Preoperative patients’ laboratory data were analyzed and the indices NLR, PLR, CAR, LCR, PNI (calculated as serum albumin (g/L) + 5× total lymphocyte count (10^9^/L)) and the mGPS (scored as 0, 1 or 2 based on CRP (>1.0 mg/dL) and albumin (<35 g/L)) were calculated. Receiver operating characteristic (ROC) analysis was used to determine the optimal cut-off for NLR, PLR, CAR, LCR, PNI in terms of distinguishing 24-month overall survival prediction. Univariate survival analysis was performed to identify prognostic factors for OS. Significant prognostic factors in univariate analysis were furthermore examined in multivariate analysis. Additionally, we analyzed the independent biomarkers for OS in selected subgroups (age ≤70 and >70 years, pT1/2 and pT3/4, pN0 and pN+).

### 2.2. Surgical Procedures

Experienced visceral surgeons with extensive experience in pancreatic surgery performed all operations, which varied depending on tumor localization. For pancreatic head tumors, the extent of local tumor involvement and the individual surgeon’s decision determined whether to resect the distal stomach (Whipple procedure) or preserve the pylorus (pylorus-preserving pancreaticoduodenectomy, PPPD). Both procedures included interaortocaval lymph node dissection; if tumor involvement was present, these were classified as pM1. In cases where intraoperative liver metastases or peritoneal carcinomatosis were evident, the primary tumor was not resected. Distal pancreatectomy always included splenectomy, and a few patients required total pancreatectomy. To achieve an R0 resection, selected cases necessitated multivisceral and/or vascular resection; however, arterial vascular resection was performed only in special cases. Intraoperative pathological examination of the pancreatic margin was conducted in all cases. If an incomplete resection (R1) was identified and it was feasible to achieve an R0 resection, further resection was performed.

### 2.3. Adjuvant Chemotherapy and Follow-Up

Postoperative chemotherapy was offered to all patients with an increasing number over the years following the introduction of general recommendation for postoperative chemotherapy. Some patients declined the treatment, while others were too impaired postoperatively to undergo chemotherapy. The choice between gemcitabine- or 5-FU-based chemotherapy was determined by the patient’s overall condition. For patients who achieved curative resection, regular follow-up with CT scans of the thorax and abdomen was recommended to occur quarterly until the third year and then every six months thereafter.

### 2.4. Statistical Analysis

Data analysis was performed with the SPSS software (IBM Corp. Released 2023. IBM SPSS Statistics for Windows, Version 29.0.2.0, Armonk, NY, USA). Comparisons of metric and ordinal data were calculated with Student’s *t*-test or Mann–Whitney U test. The Chi-square test was used for categorical data. OS was calculated for the period between the date of surgery and the date of death or last follow-up. Possible factors related to the patients’ OS were tested using univariate and multivariate analyses. Variables with a *p* ≤ 0.05 in univariate analysis were used for multivariate analysis with a Cox regression model. Survival curves were plotted using the Kaplan–Meier method and compared with the log-rank test. A *p* value ≤ 0.05 was considered statistically significant.

## 3. Results

### 3.1. Patient Characteristics, Surgical and Histopathological Details, Postoperative Course

A total of 207 patients (median age: 68 years [range 45–89 years], 46% female) were included in our analysis. The majority of patients underwent pancreatic head resection (76%), while 21% had a distal pancreatectomy and 3% a total pancreatectomy. Additional vascular resection was required in 29% of patients, while multivisceral resection needed to be performed in 17%. A R0-resection was feasible in most patients (87%). The demographical, surgical and histopathological data are shown in Table 1. A total of 48 patients showed pT1/pT2 tumors confined to the pancreas, with the largest proportion of patients (*n* = 155, 75%) showing tumor spread beyond the pancreas but without invasion of important arterial vessels (pT3); this (pT4) was the case in only a few patients (*n* = 4.2%). Local lymph node metastases (pN+) were found in the majority of cases (*n* = 123.59%), whereas distant metastasis was only found in 19 patients (9%). Most tumors (*n* = 135.64%) were poorly differentiated (G3). Postoperative morbidity occurred in 61% (127 patients), and postoperative mortality occurred in 4% (8 patients). A total of 54% of all patients received adjuvant chemotherapy (Table 1).

### 3.2. Preoperative Biomarkers

The investigated preoperative biomarkers NLR, PLR, CAR, LCR, PNI showed the identified optimal cut-off intermediate predictive values (AUC: 0.557–0.650; sensitivity: 38.7–85.6%; specificity: 35.8–78.2%) (Table 2).

### 3.3. Prognostic Factors for Overall Survival

**Figure 1:**
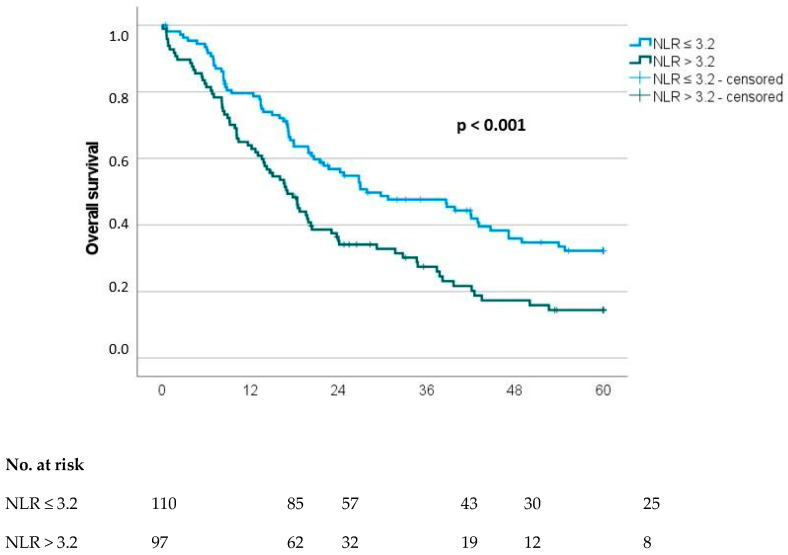
Overall survival (OS) stratified to neutrophil-to-lymphocyte ratio (NLR).

**Figure 2:**
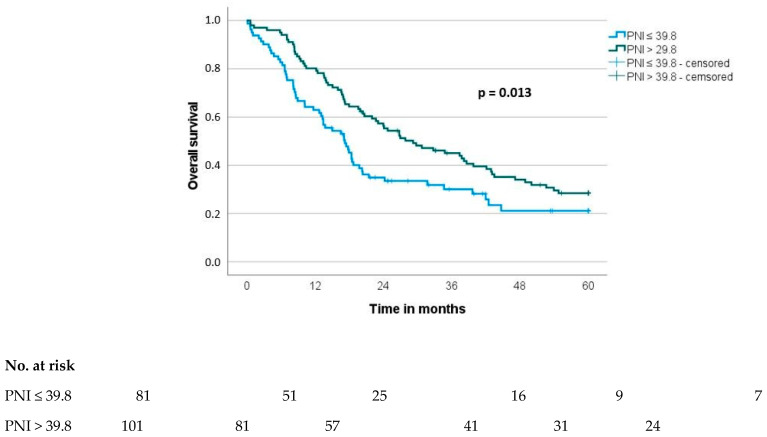
Overall survival (OS) stratified to prognostic nutritional index (PNI).

Potentially prognostic factors of patients with resected pancreatic carcinoma regarding OS are presented in Table 3. There were twelve parameters with a significant impact on overall survival in univariate analysis: age, ASA, NLR, PLR, CAR, LCR, PNI, T category, N category, M category, R classification and grading. Multivariate analysis revealed that age (hazard ratio (HR) 1.9 (1.3–2.8), *p* < 0.001), NLR (HR 1.6 (1.0–2.5), *p*= 0.048), PNI (HR 0.6 (0.4–0.9), *p* = 0.018), lymph node status (HR 2.1 (1.4–3.1), *p* < 0.001), R classification (HR 3.2 (1.8–5.9), *p* < 0.001) and grading (HR 1.6 (1.1–2.4), *p* = 0.022) were significant independent prognostic factors for OS (Table 3 and Figure 1 and Figure 2).

### 3.4. Analysis of the Independent Biomarkers NLR and PNI in Selected Subgroups

The analysis of the independent biomarkers NLR and PNI in the subgroups age ≤70 and >70 years, pT1/2 and pT3/4, pN0 and pN+ showed that the significant impact of NLR remained evident in patients with age ≤70 and with pT3/4 and pN+. Similarly, the significant impact of PNI was still observed in patients aged over 70 years, with pT3/4 and pN+ (Table 4).

## 4. Discussion

In the era of precision medicine, preoperative prognostic markers are especially valuable, as they could potentially influence treatment strategies towards an individualized therapy approach that takes more than the therapy with the best prognosis into account [20].

In this single-center evaluation of 207 patients who underwent primary pancreatic resection for PDAC, we demonstrate that NLR and PNI are independent prognostic biomarkers for OS in patients with PDAC. PDAC is characterized by an extensive tumor heterogeneity, contributing to its resilience and resulting in a wide variety of prognosis, making it a prime candidate for precision medicine [21]. In our study, we focused on different scores calculated from laboratory values typically obtained in routine preoperative lab work. This makes these scores easily accessible tools with the potential to influence therapy decisions, as they are assessed pretherapeutically. Different biomarkers have been investigated in numerous studies on various malignant tumors.

Regarding PDAC, a particular focus has been placed on the NLR [9,18,22,23,24,25,26]. In 2014, Stevens et al. performed a systematic review on the prognostic significance of the NLR in resectable PDAC [22]. Among eight studies, two showed that a low NLR was independently associated with increased survival. Due to the limited evidence, the authors called for further studies before incorporating inflammatory markers into clinical decision-making. However, a recent study by Pointer et al. investigated the NLR, PLR and lymphocyte–monocyte ratio (LMR) in 277 patients with resectable PDAC. They reported that OS and recurrence-free survival was significantly decreased in patients with an NLR ≥ 5, supporting our findings [18]. Further recent studies have mainly focused on metastatic PDAC: Toledano-Fonseca et al. combined the NLR and PLR with liquid biopsy biomarkers demonstrating that the NLR was significantly associated with OS and progression-free survival [25]. Furthermore, the NLR had a positive correlation with circulating cell-free DNA and RAS mutant allelic fraction. As oncogenic mutations in the *KRAS* gene are a hallmark of PDAC, the NLR could serve as an easy predictive tool indicating genetic alteration. Additionally, they reported that a higher NLR was associated with male age and a higher Eastern Cooperative Oncology Group (ECOG) status [27].

There is still uncertainty about the appropriate cut-off values for the NLR. Our study determined an optimal cut-off of ≤3.2, which is lower than the ≤5.52 or <5 presented by Toledano-Fonseca et al. and Pointer et al., respectively [18,25]. However, based on the current literature, a cut-off value of 2.0 to 4.0 for the NLR in PDAC patients is considered most reliable, and our cut-off falls within this range, underscoring the validity of our data [22]. Comparing the cut-off values with those of other authors, there is a noticeable trend towards lower cut-off values in the more recent literature. Considering the works up to 2014, the cut-off value here was mostly <5 to 4 [22]. Turker et al. used ≤2.17 [9], Zhou et al. ≤2.90 [24] and Huang et al. ≤3.80 [26]. Thus, our presented cut-off is in a range that has been defined by several authors and should enable valid patient discrimination. Generally speaking, a lower cut-off provides more accuracy in patient selection, whereas a too low value might support false-negative results. A robust cut-off value is therefore of utmost importance for general clinical application.

However, there are contrary results regarding the NLR: Chawla et al. focused on the NLR and PLR in patients undergoing pancreatectomy for PDAC. In their retrospective study of 217 patients, neither the NLR nor PLR were predictive for OS [16]. Similarly, Jamieson et al. conducted a prospective comparison of prognostic factors in 135 patients undergoing potentially curative surgery for PDAC and found no relationship between the NLR or PLR and OS, with most patients having a NLR (78%) and PLR (69%) within the normal range [28]. In contrast, they identified an increased modified Glasgow prognostic score (mGPS) with lower OS, which we could not confirm in our cohort. Both studies highlight significant differences in patient populations and the timing of blood samples. Since biomarker-based scores are influenced by tumor-induced changes in laboratory values, it is crucial to exclude non-tumor-related confounders such as pre-existing diseases, neoadjuvant chemotherapy, surgical interventions, and incidental infections. To address these issues, we excluded all patients receiving neoadjuvant chemotherapy, included patients with biliary obstruction only after preoperative biliary stenting, excluded patients with infections from other causes, and obtained blood samples one to three days prior to surgery. These standardizations presumably enhance the usability and comparability of our results.

In addition to the NLR, our analysis found that a PNI > 39.8, calculated from albumin and lymphocyte count, was associated with better OS. Serum albumin, also typically included in standard preoperative lab tests, not only reflects nutritional status but also plays a role in inflammation. Bullock et al. suggest that the PNI could be seen as marker of inflammation rather than nutrition [29]. Their systematic review and meta-analysis also found that the PNI was associated with OS in older adults with cancer undergoing surgery. Interestingly, they conclude that scores based on albumin and common lab tests for inflammation are useful predictors, also mentioning the mGPS. However, we did not find an association between the mGPS and OS in our patient population, which contrasts also with other studies [8].

We conducted a subgroup analysis of the LNR and PNI based on age and the T and N category to potentially identify patient groups most suited for these biomarkers. Remarkably, both independent biomarkers showed significant results in patients with a higher T category (pT3/pT4) and nodal invasion (pN+), suggesting that these biomarkers are particularly useful for assessing locally advanced PDACs. To the best of our knowledge, this finding has not been previously reported in the literature.

Our study has several limitations. The retrospective design is inherently prone to bias, including the potential bias introduced by missing data, which is a common challenge in retrospective analyses. However, in our study, the rate of missing data does not exceed 15% for any parameter, which may be considered acceptable. The long study period of 18 years, encompassing changes and improvements in therapy, combined with a limited patient cohort, affects the quality of our data. Additionally, inflammatory scores can be influenced by factors other than tumor development, and it may not be possible to exclude all confounding factors with certainty retrospectively. Furthermore, we did not record specific chemotherapy regimens for adjuvant chemotherapy or patients’ preoperative conditions (e.g., ECOG status), which could significantly influence patient outcomes.

## 5. Conclusions

In conclusion, the NLR and PNI could be helpful tools for prediction of prognosis in patients with PDAC undergoing pancreatic resection in curative intention, especially in locally advanced tumors. However, conflicting results still exist, which emphasizes the need for further prospective studies.

## Figures and Tables

**Table 1:** Characteristics of patients undergoing primary resection for pancreatic ductal adenocarcinoma.

Patient Characteristics	*n* = 207
Demographic characteristics	
Age (years), median (IQR)	68 (15)
Gender (female/male), *n* (%)	95 (46)/112 (54)
ASA (I/II/III) (*n* = 196) *, *n* (%)	4 (2)/121 (62)/71 (36)
BMI (kg/m^2^) (*n* = 203) *, median (IQR)	25.6 (4.9)
Alcohol abuse, *n* (%)	98 (47)
Nicotine abuse, *n* (%)	45 (22)
Comorbidity, *n* (%)	
Hypertension	113 (55)
Diabetes	58 (28)
Cardiovascular	25 (12)
Pulmonary	19 (9)
Cerebrovascular	12 (6)
Liver disease	16 (8)
Preoperative biliary stenting, *n* (%)	107 (52)
Preoperative CA19-9 (U/mL) (*n* = 186) *, median (IQR)	94 (391)
Surgical characteristics	
Kind of surgery (Pancreatic head resection/Distal pancreatectomy/Total pancreatectomy), *n* (%)	157 (76)/43 (21)/7 (3)
Vascular resection, *n* (%)	59 (29)
Multivisceral resection, *n* (%)	36 (17)
Operative time (min), median (IQR)	283 (105)
Intraoperative blood transfusion, *n* (%)	56 (27)
Histopathological characteristics	
T category (pT1/pT2/pT3/pT4), *n* (%)	12 (6)/36 (17)/155 (75)/4 (2)
N category (pN0/pN+), *n* (%)	84 (41)/123 (59)
M category (pM0/pM1), *n* (%)	188 (91)/19 (9)
R classification (R0/R1/2), *n* (%)	181 (87)/26 (13)
Grading (G1/G2/G3), *n* (%)	4 (2)/69 (33)/134 (65)
Postoperative characteristics	
Postoperative morbidity, *n* (%)	127 (61)
Postoperative mortality, *n* (%)	8 (4)
Adjuvant chemotherapy, *n* (%)	111 (54)

ASA = American Society of Anesthesiologists classification; BMI = body mass index; * missing data.

**Table 2:** Optimal cut-offs for preoperative biomarkers to predict 24-month overall survival using ROC analysis (*n* = 207).

Preoperative Biomarkers	Median (IQR)	ROC Analysis			
		Optimal Cut-Off	AUC	Sensitivity	Specifity
NLR (*n* = 207) *	3.1 (2.1)	≤3.2	0.610	56.3%	64.8%
PLR (*n* = 206) *	172 (115)	≤222	0.557	38.7%	78.2%
CAR (*n* = 180) *	0.14 (0.46)	≤0.05	0.588	85.6%	35.8%
LCR (*n* = 200) *	0.26 (0.60)	≤0.22	0.575	65.5%	51.7%
PNI (*n* = 182) *	40.5 (7.1)	≤39.8	0.650	70.4%	55.4%
mGPS (*n* = 181) *, *n* (%)	110 (61)/43 (24)/28 (16)	-	-	-	-

NLR = neutrophil-to-lymphocyte ratio; PLR = platelet-to-lymphocyte ratio; CAR = CRP-to-albumin ratio; LCR = lymphocyte-to-CRP ratio; PNI = prognostic nutritional index (10× serum albumin + 5× lymphocytes); mGPS = modified Glasgow prognostic score; * missing data.

**Table 3:** Prognostic factors of patients with resected pancreatic ductal adenocarcinoma for overall survival (OS) (*n* = 207).

			Overall Survival (OS)				
			Univariate		Multivariate		
		*n*	Median OS	*p*	HR	95% CI	*p*-Value
Age	≤70 years	120	29.2	**<0.001**	1.9	1.3–2.8	**<0.001**
	>70 years	87	17.1				
Gender	Female	95	27.0	0.104			
	Male	112	18.7				
ASA	I	4	-	**0.027**	1.0	0.5–32.8 0.7–43.2	0.187 0.116
	II	121	24.0		4.1		
	III	71	17.0		5.3		
BMI	≤20 kg/m^2^	16	13.4	0.624			
	>20 and ≤25 kg/m^2^	73	24.0				
	>25 and ≤30 kg/m^2^	79	19.9				
	>30 kg/m^2^	35	24.2				
CA19-9	≤37 U/mL	58	24.8	0.251			
	>37 U/ml	128	20.7				
NLR	≤3.2	110	27.9	**<0.001**	1.6	1.0–2.5	**0.048**
	>3.2	97	17.1				
PLR	≤222	141	24.8	**0.021**	1.0	0.6–1.6	0.973
	>222	65	18.4				
CAR	≤0.05	43	38.2	**0.013**	1.4	0.8–2.3	0.250
	>0.05	137	18.0				
LCR	≤0.22	89	17.2	**0.017**	1.3	0.8–2.0	0.309
	>0.22	111	27.0				
PNI	≤39.8	81	17.2	**0.013**	0.6	0.4–0.9	**0.018**
	>39.8	101	29.2				
mGPS	0	110	24.0	0.105			
	1	43	27.9				
	2	28	13.4				
Kind of surgery	Pancreatic head resection	157	24.0	0.145			
	Distal pancreatectomy	43	18.0				
	Total pancreatectomy	7	6.7				
Vascular resection	Yes	59	17.2	0.209			
	No	148	22.7				
Multivisceral resection	Yes	36	17.0	0.133			
	No	171	23.8				
T category	pT1/pT2	48	37.8	**0.013**	1.6	1.0–2.7	0.054
	pT3/pT4	159	18.4				
N category	pN0	84	39.8	**<0.001**	2.1	1.4–3.1	**<0.001**
	pN+	123	17.3				
M category	M0	188	23.8	**0.014**	0.8	0.4–1.7	0.588
	pM1	19	12.4				
R classification	R0	181	24.0	**<0.001**	3.2	1.8–5.9	**<0.001**
	R1/R2	26	8.4				
Grading	G1/G2	73	37.8	**<0.001**	1.6	1.1–2.4	**0.022**
	G3	134	17.2				
Morbidity	Yes	127	19.8	0.379			
	No	80	29.2				
Adjuvant chemotherapy	Yes	111	23.8	0.311			
	No	96	17.5				

ASA = American Society of Anesthesiologists classification; BMI = body mass index; NLR = neutrophil-to-lymphocyte ratio; PLR = platelet-to-lymphocyte ratio; CAR = CRP-to-albumin ratio; LCR = lymphocyte-to-CRP ratio; PNI = prognostic nutritional index (10× serum albumin + 5× lymphocytes); mGPS = modified Glasgow prognostic score.

**Table 4:** Analysis of the identified independent biomarkers for overall survival in selected subgroups.

Subgroups		Biomarker		*n*	Median OS	*p*
Age	≤70 years	NLR	≤3.2	66	42.9	**0.005**
			>3.2	54	19.8	
		PNI	≤39.8	41	21.5	0.659
			>39.8	63	37.4	
	>70 years	NLR	≤3.2	43	19.9	0.081
			>3.2	44	13.8	
		PNI	≤39.8	40	8.6	**<0.001**
			>39.8	38	23.1	
T category	pT1/pT2	NLR	≤3.2	23	-	0.111
			>3.2	25	34.7	
		PNI	≤39.8	21	34.7	0.469
			>39.8	20	38.2	
	pT3/pT4	NLR	≤3.2	86	26.8	**<0.001**
			>3.2	72	14.0	
		PNI	≤39.8	60	13.3	**0.006**
			>39.8	81	26.8	
N category	pN0	NLR	≤3.2	50	43.1	0.656
			>3.2	33	37.8	
		PNI	≤39.8	32	21.5	0.202
			>39.8	45	42.9	
	pN+	NLR	≤3.2	59	24.8	**<0.001**
			>3.2	64	13.6	
		PNI	≤39.8	49	12.7	**0.032**
			>39.8	56	22.0	

NLR = neutrophil-to-lymphocyte ratio; PNI = prognostic nutritional index (10× serum albumin + 5× lymphocytes).

## Data Availability

All data have been included in the manuscript and the tables.

## Abbreviations

CAR	CRP–albumin ratio
CRP	C-reactive protein
ECOG	Eastern Cooperative Oncology Group
LCR	Lymphocyte–CRP ratio
mGPS	Modified Glasgow prognostic score
NLR	Neutrophil–lymphocyte ratio
OS	Overall survival
PDAC	Pancreatic ductal adenocarcinoma
PLR	Platelet–lymphocyte ratio
PNI	Prognostic nutritional index
PO	Precision oncology
UICC	Union for International Cancer Control
